# Assessing the impact of booster vaccination on diphtheria transmission: Mathematical modeling and risk zone mapping

**DOI:** 10.1016/j.idm.2024.01.004

**Published:** 2024-01-17

**Authors:** Ilham Saiful Fauzi, Nuning Nuraini, Ade Maya Sari, Imaniah Bazlina Wardani, Delsi Taurustiati, Purnama Magdalena Simanullang, Bony Wiem Lestari

**Affiliations:** a Department of Accounting, Politeknik Negeri Malang, Malang, Indonesia; b Department of Mathematics, Faculty of Mathematics and Natural Sciences, Institut Teknologi Bandung, Bandung, Indonesia; c Center for Mathematical Modeling and Simulation, Institut Teknologi Bandung, Bandung, Indonesia; d Study Program of Biology Education, Faculty of Education and Teacher Training, UIN Kiai Haji Achmad Siddiq Jember, Jember, Indonesia; e West Java Provincial Health Office, West Java, Indonesia; f Department of Public Health, Faculty of Medicine, Universitas Padjadjaran, Bandung, Indonesia; g Department of Internal Medicine, Radboud Institute for Health Sciences, Radboud University Medical Centre, Nijmegen, the Netherlands

**Keywords:** Diphtheria, Vaccination, Mathematical modeling, SIR model, Hot spot analysis, 00–01, 99-00

## Abstract

The COVID-19 pandemic caused significant disruptions in the healthcare system, affecting vaccinations and the management of diphtheria cases. As a consequence of these disruptions, numerous countries have experienced a resurgence or an increase in diphtheria cases. West Java province in Indonesia is identified as one of the high-risk areas for diphtheria, experiencing an upward trend in cases from 2021 to 2023. To analyze the situation, we developed an SIR model, which integrated DPT and booster vaccinations to determine the basic reproduction number, an essential parameter for infectious diseases. Through spatial analysis of geo-referenced data, we identified hotspots and explained diffusion in diphtheria case clusters. The calculation of *R*_0_ resulted in an *R*_0_ = 1.17, indicating the potential for a diphtheria outbreak in West Java. To control the increasing cases, one possible approach is to raise the booster vaccination coverage from the current 64.84% to 75.15%, as suggested by simulation results. Furthermore, the spatial analysis revealed that hot spot clusters were present in the western, central, and southern regions, posing a high risk not only in densely populated areas but also in rural regions. The diffusion pattern of diphtheria clusters displayed an expansion-contagious pattern. Understanding the rising trend of diphtheria cases and their geographic distribution can offer crucial insights for government and health authorities to manage the number of diphtheria cases and make informed decisions regarding the best prevention and intervention strategies.

## 1 Introduction

Diphtheria, a highly contagious and potentially deadly bacterial infection, has been a concern for public health worldwide. This infectious disease is caused by the bacterium *Corynebacterium diphtheriae* and primarily affects the respiratory system, leading to the formation of a thick greyish-white membrane in the throat and nasal passages (Rakhmanova et al., 2019). Diphtheria spreads through respiratory droplets, making it easily transmissible from person to person, especially in crowded and unhygienic environments. Annually, 5,000 cases of diphtheria were documented on a global scale (Zakikhany & Efstratiou, 2012). However, in the year 2017, the worldwide reported cases surged to a total of 8,819, marking the highest count since 2004 (Clarke et al., 2019). Within the period of 2016–2019, multiple countries, such as Bangladesh, Yemen, Venezuela, and Haiti, experienced outbreaks of diphtheria (Badell et al., 2021; Lodeiro-Colatosti et al., 2018; Rahman & Islam, 2019). These outbreaks often affected vulnerable populations or areas that faced social disruption and conflict. Indonesia is one of the risk areas for diphtheria and encountered a significant public health concern as diphtheria incidence escalated from 2010 and persisted in its rise until 2017 (Karyanti et al., 2019).

It is generally recognized that the most effective method to control diphtheria is through vaccination. The diphtheria vaccine, often administered as a combination vaccine along with pertussis and tetanus (DPT), has played a pivotal role in reducing the incidence of this disease globally (Skibinski et al., 2011). As an additional measure to sustain protection, a booster vaccination is recommended during adolescence or adulthood. Regular and booster immunization not only protects individuals from contracting diphtheria but also helps to create herd immunity, shielding vulnerable populations who cannot receive the vaccine. Vaccination coverage has increased significantly over the last 37 years. In 1974, less than 5% of infants worldwide were completely immunized, whereas, by 2005, the global coverage for the third dose of the DPT vaccine had risen to 79% (Kumar & Mohanty, 2011). In 2010, more than two-thirds of countries in the world had achieved 90% DPT coverage, and an estimated 85% of infants globally had received at least 3 doses of vaccine (Centers for Disease Control and Prevention, 2011).

The onset of the COVID-19 pandemic in recent times has brought about unprecedented challenges to global health systems. While the focus has been primarily on controlling and managing the COVID-19 virus, there have been indirect consequences on other infectious diseases, including diphtheria. Disruptions in healthcare services, lockdown measures, and shifts in priorities have impacted routine immunization programs, leading to a decline in diphtheria vaccination coverage in some areas (Lassi et al., 2021). This has created favorable conditions for the resurgence of diphtheria cases in certain regions, as a weakened vaccination coverage leaves populations susceptible to preventable diseases. Numerous studies around the world have shown a re-emergence of diphtheria cases and an increasing trend of cases after the pandemic, such as in Pakistan (Saeed et al., 2023), India (World Health Organization, 2023), Peru (Mezones-Holguin et al., 2021), and Nigeria (Ibrahim et al., 2022).

West Java province is situated in the central-southern part of Indonesia, neighboring the capital city, Jakarta. It has an approximate population of 50 million, with over 75% residing in urban and semi-urban regions. West Java stands out as one of the high-risk areas in Indonesia for diphtheria infection, primarily due to its concentration of large cities with dense populations. The region's urban centers, distributed in the central, northwestern, and northeastern regions, witness significant population influxes, resulting in close living quarters and increased opportunities for disease transmission. The incidence of diphtheria in West Java fluctuated from 2010 to 2017, where in 2016 there was a significant increase of 3 times compared to the previous period accompanied by an increase in the case fatality rate (CFR) (Harapan et al., 2019). Through increasing vaccination coverage in all districts/cities, the number of diphtheria cases has been reduced gradually until 2020 with low lethal risk. Unfortunately, disruptions to health services during the COVID-19 pandemic led to an unexpected increase in diphtheria cases during the period January 2021 to March 2023 as reported by the West Java Provincial Health Office (West Java Provincial Health Office, 2023).

Therefore, this study aimed two folds, first to assess the effectiveness of booster vaccination in controlling diphtheria transmission through mathematical modelling, and second, to provide a comprehensive analysis of the spatial distribution of diphtheria cases, including identifying clusters and hotspots where the infection is more prevalent in West Java. Furthermore, this research will inform relevant stakeholders in designing targeted interventions and resource allocation to effectively combat diphtheria outbreaks and protect vulnerable communities, fostering a safer and healthier environment in West Java.

## 2 Material and methods

### 2.1 Setting and data collection

COVID-19 has caused disruptions in vaccination programs globally, including Indonesia (Basu et al., 2023; Harris et al., 2021; Kusumaningrum et al., 2022). Insufficient immunization coverage, limited access to healthcare services, and a lack of public awareness were contributing factors to the outbreak of some preventable diseases, including diphtheria. During COVID-19, health resources and attention have been diverted towards addressing the pandemic, potentially impacting the surveillance, diagnosis, and management of diphtheria cases.

**Fig. 1:**
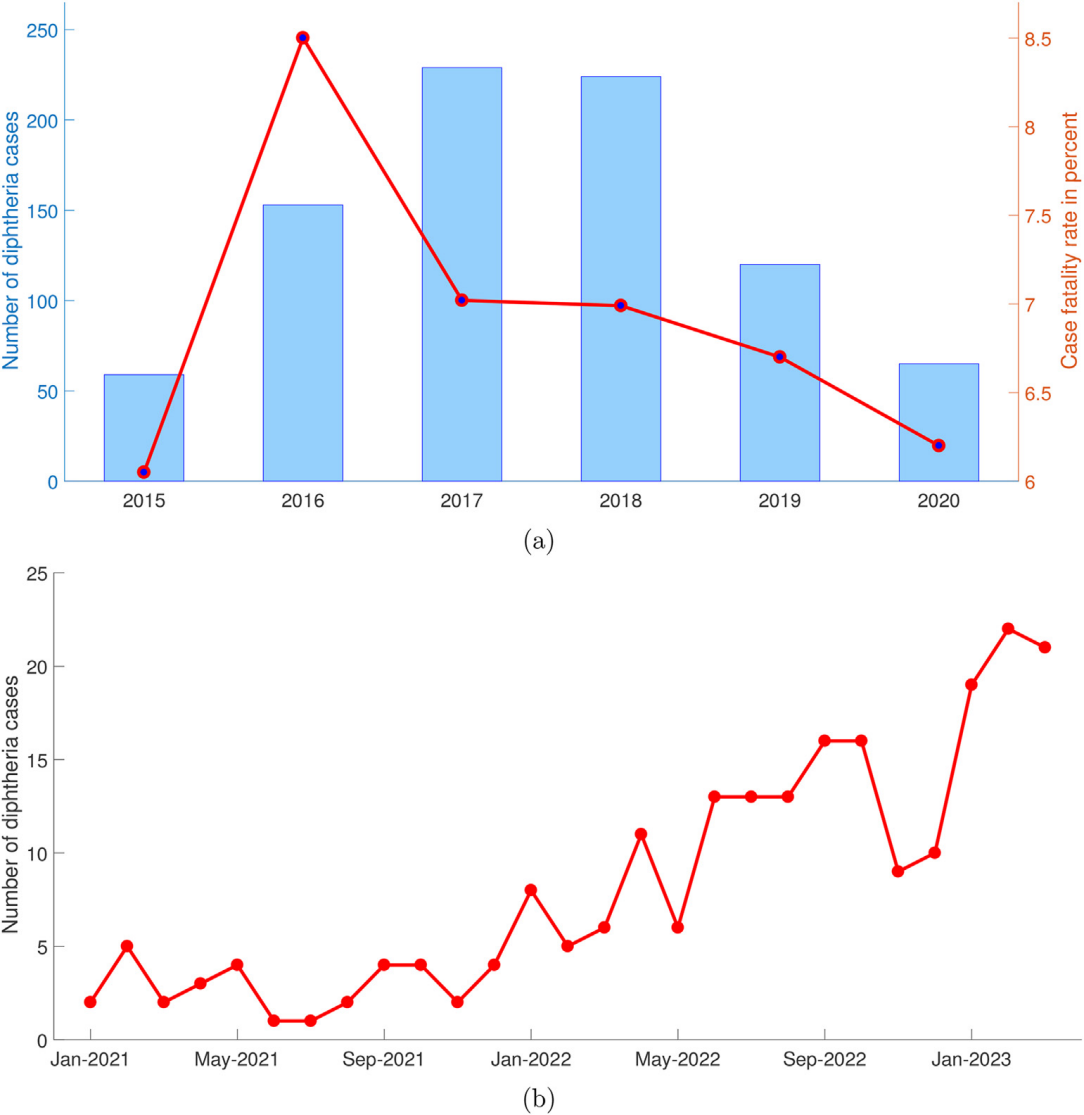
Annual number of diphtheria cases and CFR in West Java during 2015–2020 are shown in (a), and monthly number of diphtheria cases in West Java after COVID-19 pandemic was shown in (b).

West Java province is considered one of the high-risk areas for diphtheria infection in Indonesia due to its densely populated human population. With a significant number of people living in close proximity, the potential for transmission of the diphtheria bacteria increases. The close quarters and crowded living conditions can facilitate the spread of the disease, especially in areas with limited access to healthcare services and lower vaccination coverage. Based on the health profile compiled by the West Java Provincial Health Office, the number of diphtheria cases increased significantly in 2016 and has tended to decrease slowly until 2020, accompanied by a decrease in the case fatality rate (CFR) (West Java Provincial Health Office, 2020). Fig. 1a shows the number of diphtheria cases and CFR during 2015–2020 period. Unfortunately, diphtheria cases in various areas in West Java tend to show an increasing trend after the COVID-19 pandemic. As can be seen in Fig. 1b, the monthly case accumulation trend in West Java is increasing from January 2021 to April 2023 (West Java Provincial Health Office, 2023).

To protect individuals from diphtheria infection, a combination vaccine called DPT, comprising Diphtheria, Pertussis (whooping cough), and Tetanus antigens, is administered in a series of three doses (Liang et al., 2018). DPT vaccination stimulates the body's immune system to produce antibodies against the causative bacteria. By administering this vaccine to infants and young children, it is expected that the number of diphtheria cases can be significantly reduced. DPT 1 is typically given during infancy, where the vaccine introduces harmless components of the diphtheria bacterium into the body (Burton et al., 2009). This exposure triggers the immune system to develop specific antibodies against diphtheria toxins. After a few weeks, DPT 2 is administered to reinforce the immune response, further strengthening the protection against diphtheria (Burton et al., 2009). Subsequently, DPT 3 is given a few months later, completing the primary series, and ensuring a robust and lasting immunity (Awofeso et al., 2013).

As an additional measure to sustain protection, a booster vaccination is recommended after a few years. Booster vaccinations for diphtheria are typically recommended during adolescence or adulthood, with periodic updates throughout an individual's life (Hofmann, 2005). The booster contains the diphtheria antigen, reminding the immune system of its initial training and maintaining the ability to rapidly respond to any potential diphtheria exposure. The combination of DPT 1, DPT 2, DPT 3, and the booster vaccination provides long-term immunity and a solid defense against diphtheria, safeguarding individuals and contributing to the overall public health by reducing the risk of diphtheria transmission.

**Fig. 2:**
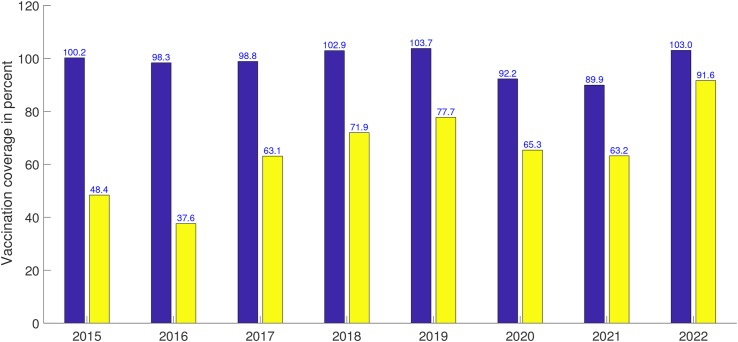
DPT (blue) and booster (yellow) vaccination coverage in West Java during 2015–2022.

In recent years, West Java province has improved public health service related to diphtheria through comprehensive vaccination programs. With the introduction of the DPT vaccine, the incidence of diphtheria has decreased in every regions in West Java. West Java province faces challenges in maintaining high vaccination coverage across all regions due to geographic disparities, cultural beliefs, and limited healthcare access. However, the coverage of DPT and booster vaccinations is relatively high in the last 8 years. The coverages of DPT and booster vaccination in West Java were presented in Fig. 2.

### 2.2 Mathematical model

In order to elucidate the mechanism of diphtheria transmission in the human population, we used the classic SIR model integrated with the vaccination process. The SIR model is a widely used mathematical framework in epidemiology to study the spread of infectious diseases within a population (Chen, 2014 ). It provides a mathematically rigorous and well-defined framework to study disease dynamics, enabling the formulation of differential equations that accurately represent the flow of individuals in each compartment.

**Fig. 3:**
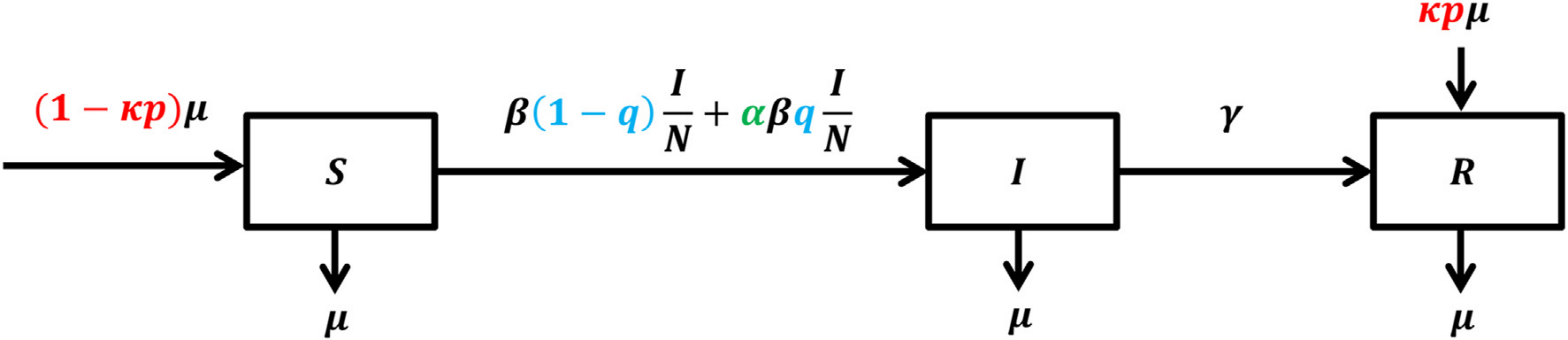
A schematic diagram of the diphtheria transmissions within human populations.

In our proposed model, the population is classified into three epidemiological classes: susceptible (*S*), infected (*I*), and recovered (*R*). The compartment *S* represents individuals who are susceptible to the diphtheria infection but have not yet been infected. The compartment *I* comprises individuals who are currently infected and capable of spreading the diphtheria bacteria to susceptible individuals. The compartment *R* consists of individuals who have recovered from the diphtheria infection. The scheme of the mathematical model showing the diphtheria transmission process is presented in Fig. 3.

We assumed that three doses of DPT vaccination given to newborns and children with age less than 1 year were complete vaccinations that induced immunity from diphtheria infection. The proportion of DPT vaccination in newborns and young children is *p*. The probability of success in preventing diphtheria is expressed by *κ*. Therefore the recruitment rate for the susceptible compartment is (1 − *κp*)*μ*, where *μ* represents the human natural birth rate. Meanwhile, the proportion of *κpμ* directly enters the recovery compartment, which is immune to infection and demonstrates the success of complete DPT vaccination to prevent diphtheria infection. Booster vaccinations are administered to individuals in the adult population (aged over 1 year), with a coverage proportion denoted as *q*, representing the fraction of the susceptible population receiving the booster dose. Consequently, the remaining susceptible individuals, constituting a proportion of 1 − *q*, are left vulnerable to diphtheria infection if they encounter infected individuals at a transmission rate *β*. The susceptible population with a proportion *q* receiving boosters vaccination for diphtheria is still at risk of infection, albeit at a lower infection rate *αβ* with 0 < *α* < 1. The parameter *γ* indicates the recovery rate of human from diphtheria infection, progressing from compartment *I* to compartment *R*. Additionally, all compartments decrease at a rate *μ* which represents the natural human mortality rate.

The dynamics of individuals in each epidemiological state are described by the following set of nonlinear ordinary differential equations:

(1) $$\begin{array}{ll} & \frac{dS}{dt}=\left(1-\kappa p\right)\mu N-\left[\beta \left(1-q\right)+\alpha \beta q\right]\frac{SI}{N}-\mu S\\ & \frac{dI}{dt}=\left[\beta \left(1-q\right)+\alpha \beta q\right]\frac{SI}{N}-\left(\gamma +\mu \right)I\\ & \frac{dR}{dt}=\kappa p\mu N+\gamma I-\mu R \end{array}$$

We considered a constant human population, *N*=*S* + *I* + *R*. By substituting the fact that *S*=*N* − (*I* + *R*), we obtain the two-dimensional equations as follows:

(2) $$\begin{array}{ll} & \frac{dI}{dt}=\left[\beta \left(1-q\right)+\alpha \beta q\right]\left(N-\left(I+R\right)\right)\frac{I}{N}-\left(\gamma +\mu \right)I\\ & \frac{dR}{dt}=\kappa p\mu N+\gamma I-\mu R \end{array}$$

The differential equation system has two possible equilibriums. The first equilibrium is the disease-free equlibrium (DFE), (*I*°, *R*°) = (0, *κpN*). The next equilibrium is the endemic equilibrium (EE) given by:

(3) $$\left(I^{*},R^{*}\right)=\left(\frac{\mu \left(\delta \left(1-\kappa p\right)-\left(\mu +\gamma \right)\right)}{\delta \left(\mu +\gamma \right)}N,\frac{\delta \left(\kappa p\mu +\gamma \right)-\gamma \left(\mu +\gamma \right)}{\delta \left(\mu +\gamma \right)}N\right)$$

where *δ* = *β*(1 − *q* + *αq*).

The calculation of the basic reproduction number, denoted as *R*_0_, was accomplished through the application of the Next Generation Matrix (NGM). By considering the infected subsystem and implementing linearization at the disease-free equilibrium, a Jacobian matrix was obtained. The complete procedure for calculating the *R*_0_ through the NGM method can be seen in Appendix .1. The basic reproduction number was then derived from the dominant eigenvalue of the Jacobian matrix and shown as follows:

(4) $$R_{0}=\frac{\beta \left(1-q+\alpha q\right)\left(1-\kappa p\right)}{\gamma +\mu }$$

The existence of the disease-free equilibrium is guaranteed. However, the existence of endemic equilibrium requires additional condition. Parameters *p* and *q* represent the proportion of DPT and booster vaccination coverages respectively, so we have 0 < *p*, *q* < 1. By using this condition, we obtain *δ* > 0. Hence, we require the condition *R*_0_ > 1 for the existence of endemic equilibrium. The stability analysis at disease-free equilibrium (DFE) and endemic equilibrium (EE) are presented in Theorems shown in Appendix .2.

Furthermore, the model includes sensitivity analysis for *R*_0_, aiming to identify the most influential parameter. Sensitivity index of *R*_0_, which relies on a user-defined parameter *y* and is represented by Eq. (5), illustrates the extent of *R*_0_ variations with changes in each parameter. The evaluation of sensitivity indices using the estimated values for *β*, *α*, and *κ* is presented in results section.

(5) $$S_{y}^{R_{0}}=\frac{\partial R_{0}}{\partial y}\times \frac{y}{R_{0}}$$

For the numerical simulation, the value of unobserved parameters (*β*, *α*, *κ*) are estimated by minimizing the root-mean-square error (RMSE) that measures the average difference between a mathematical model's predicted values and the actual diphtheria data. The Spiral Dynamic Optimization (SDO) method (Tamura & Yasuda, 2011) with 1000 random points and 50 iterations is carried out by selecting the best value of parameter (*β*, *α*, *κ*). Meanwhile, values for other parameters were obtained from either literature studies or estimated from the data. According to the assumptions we mentioned before, the parameter *p* and *q* represent the proportion of DPT and booster vaccination coverage with a value of 0 < *p*, *q* < 1. As can be seen in Fig. 2, some DPT vaccination coverage in the data has a value of more than 100%. Hence, we use the average value of the data for the 2015–2022 period to obtain the parameter value of *p* and *q*. Parameter descriptions and respective values are summarized in Table 1.

**Table 1:** Description of variables and parameters used in mathematical model.

Parameter	Description	Value	Unit	References
*μ*	Human natural birth and mortality rate	1/(65 ⋅ 12)	month^−1^	(Nuraini et al., 2021; Sornbundit et al., 2017)
*γ*	Recovery rate of the infected people	1/2	month^−1^	(Ghani et al., 2023; Kanchanarat et al., 2022)
*p*	Proportion of DPT vaccination coverage	98.62%	–	data
*q*	Proportion of booster vaccination coverage	64.84%	–	data
*N*	Total population in West Java province	5 ⋅ 10^7^	people	Fauzi et al. (2022)

### 2.3 Risk zone mapping

At first, we examined the existence of an overall spatial cluster of annual diphtheria cases in West Java province by determining Moran's Index. Spatial association analysis using Moran's Index is a useful method for assessing the clustering or dispersion of spatial patterns in geographic data (Fauzi et al., 2022). It is widely used in fields such as geography (Lee & Li, 2017), ecology (Legendre & Fortin, 1989), urban planning (Wang et al., 2023), and epidemiology (Gebreab et al., 2018). The Moran's Index quantifies the degree of spatial autocorrelation, indicating whether similar values tend to be close to each other or dispersed across the study area. A positive Moran's Index suggests spatial clustering, where similar values are concentrated together, while a negative index indicates spatial dispersion (Li et al., 2007). The value of Moran's Index (Moran, 1950) can obtained by implementing the following formula:

(6) $$I=\frac{n\sum_{i}\sum_{j}w_{ij}\left(x_{i}-\bar{x}\right)\left(x_{j}-\bar{x}\right)}{\sum_{i}\sum_{j}w_{ij}\sum_{i}{\left(x_{i}-\bar{x}\right)}^{2}}$$

with *w*_*ij*_ is spatial weight between feature *i* and *j*. If region *i* is adjacent with region *j*, then *w*_*ij*_ = 1, otherwise *w*_*ij*_ = 0. Variable *x*_*j*_ indicates the number of cases, *n* is the number of areas in study region, and $\bar{x}$ denotes the mean of diphtheria cases data.

Following the identification of potential spatial clustering of diphtheria cases, we proceeded to investigate these clusters at a more localized level to identify hot spot areas, offering valuable insights into the spatial aspects of diphtheria transmission. A hot spot analysis is a valuable tool for identifying clusters and assessing their significance within our dataset. These significant clusters, referred to as hot spots, indicate areas with an increased risk of diphtheria infections. Among the commonly used statistical measures for hot spot analysis is the $G_{i}^{*}$ statistic, also known as the Getis-Ord $G_{i}^{*}$ statistic (Getis & Ord, 2010). This statistic is calculated using a formula (Getis & Ord, 2010; Ord & Getis, 1995) as follows:

(7) $$G_{i}^{*}=\frac{\sum_{j}w_{ij}x_{j}}{\sum_{j}x_{j}}$$

The $G_{i}^{*}$ statistic measures the degree of association between regions within the study area. The greater the $G_{i}^{*}$ value, the higher the significance of an area. Further, we computed the corresponding Z-score for each year using the diphtheria data as the observed variable. The calculation of the value of $Z_{G_{i}^{*}}$ (Getis & Ord, 2010; Ord & Getis, 1995) is given as follows:

(8) $$Z_{G_{i}^{*}}=\frac{\sum_{j}w_{ij}x_{j}-\left(\sum_{j}w_{ij}\right)\left(\bar{x}\right)}{s\sqrt{\frac{n\sum_{j}w_{ij}^{2}-{\left(\sum_{j}w_{ij}\right)}^{2}}{n-1}}}$$

with *n* = 27 that indicates the number of areas including city or district in West Java. Moreover, $\bar{x}$ and *s* denote mean and variance, respectively.

$$\bar{x}\left(i\right)=\frac{\sum_{j}x_{j}}{n}\;\;\;s^{2}\left(i\right)=\frac{\sum_{j}x_{j}^{2}}{n}-{\left[\bar{x}\left(i\right)\right]}^{2}$$

The statistic provided in Eq. (8) assesses the spatial arrangement of geo-referenced data by considering both the feature values and their corresponding locations together, focusing on a local viewpoint. This enables the detection of hotspots and cold spots in the data. Based on the value of Z-score, each area was categorized using colors. A hot spot with a $Z_{G_{i}^{*}}>2$ was represented by the color brown and identified as high-level hot spot, while an area with a $1<Z_{G_{i}^{*}}\leq 2$ was denoted by the color orange and classified as moderate-level hot spot. Areas with a $0<Z_{G_{i}^{*}}\leq 1$ were assigned the color beige and identified as low-level hot spot. If the Z-score fell below 0, the area was colored white, indicating it was not classified as a hot spot and we labeled it as cold spot. The resulting hot spot map effectively highlights clusters of areas that require immediate attention.

**Fig. 4:**
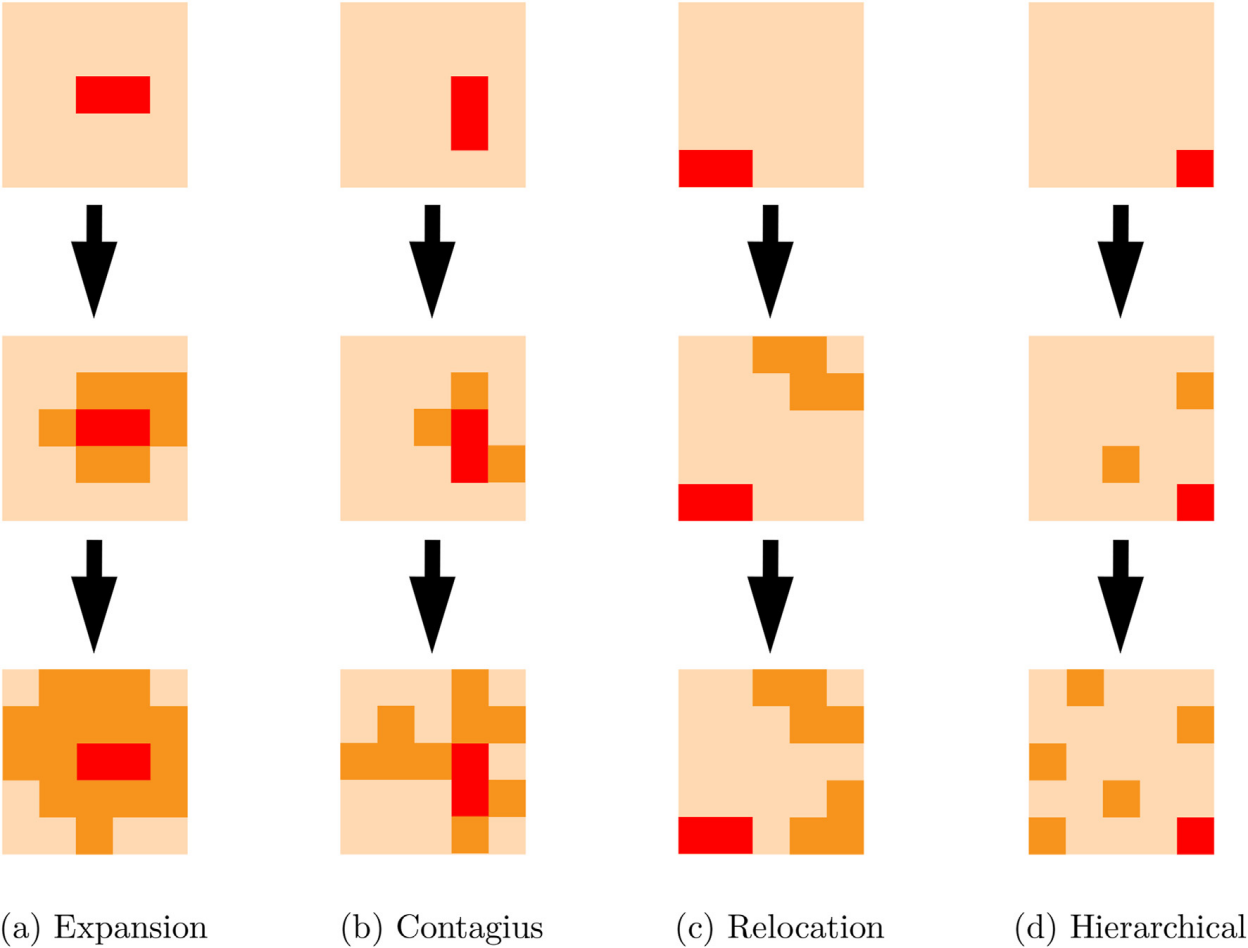
Diffusion pattern of spatial phenomenon starting with red regions as the initial hot spot.

In addition, we identified the spatial diffusion of diphtheria transmission in West Java province. Fig. 4 shows an illustration of the spatial diffusion pattern. Hornsby (Hornsby, 2011) introduced four distinct patterns of spatial diffusion as follows:

1. **Expansion diffusion**: the growth of the spatial phenomenon is characterized by its simultaneous expansion in all directions.
2. **Contagius diffusion**: the spread of the spatial phenomenon from one location to nearby locations, creating a ripple effect of transmission.
3. **Relocation diffusion**: the process of dispersal involves moving to a different region where the potential dispersal pattern might differ.
4. **Hierarchical diffusion**: the spatial phenomenon spreads through a hierarchical arrangement of locations, such as an urban hierarchy.

## 3 Results

In this section, we described the diphtheria cases that have occurred in West Java province which shows an indication of an increase in cases after the COVID-19 pandemic. The summary of diphtheria cases in West Java after pandemic is shown in Table 2. Also, we presented the numerical simulation results of our proposed mathematical model by estimating the parameters that minimize the error between the data and the model output. The model output refers to the simulation results in infected compartment (*I*) which shows the number of individuals infected with diphtheria. We examined how a booster vaccination strategy can effectively reduce the risk of increased cases. In addition, we also spatially analyzed the spread of diphtheria cases by identifying hot spots and classifying the diffusion pattern based on annual data for the 2021–2023 period (after pandemic).

**Table 2:** Summary of the diphtheria cases recorded in West Java after the COVID-19 pandemic.

	2021	2022	2023
	January–December	January–December	January–March
Total cases	34	125	63
Sex			
Male	17 (50%)	65 (52%)	30 (48%)
Female	17 (50%)	60 (48%)	33 (52%)
Age group			
0–10 years	18 (53%)	73 (58%)	27 (43%)
10–20 years	3 (9%)	16 (13%)	16 (25%)
20–30 years	7 (20%)	18 (14%)	11 (18%)
30–40 years	4 (12%)	11 (9%)	5 (8%)
>40 years	2 (6%)	7 (6%)	4 (6%)
Vaccination status			
Complete	6 (18%)	35 (28%)	11 (17%)
Partial	0 (0%)	11 (8%)	9 (14%)
Never	3 (9%)	32 (26%)	23 (37%)
No information	25 (73%)	47 (38%)	20 (32%)
Fever			
Yes	19 (56%)	107 (86%)	58 (89%)
No	4 (12%)	14 (11%)	4 (6%)
No information	11 (32%)	4 (3%)	1 (2%)
Sore throat			
Yes	22 (65%)	110 (88%)	56 (89%)
No	0 (0%)	12 (10%)	7 (11%)
No information	12 (35%)	3 (2%)	0 (0%)
Bullneck			
Yes	13 (38%)	56 (45%)	19 (30%)
No	8 (24%)	64 (51%)	44 (70%)
No information	13 (38%)	5 (4%)	0 (0%)
Pseudomembrane			
Yes	21 (62%)	93 (75%)	45 (71%)
No	2 (6%)	23 (18%)	12 (19%)
No information	11 (32%)	9 (7%)	6 (10%)

### 3.1 Description of diphtheria cases in West Java

A detailed description of diphtheria cases in a period of 2021–2023 is depicted in Table 2 (West Java Provincial Health Office, 2023). In 2021, a year after COVID-19 pandemic, West Java Provincial Health Office reported 34 cases of diphtheria distributed throughout the region. A notable surge in diphtheria cases was reported in West Java in 2022, where the incidence nearly quadrupled compared to the previous year. A total of 125 cases were detected across several districts and cities. During the initial three-month period in 2023, the reported instances of diphtheria have already reached fifty percent of the total cases recorded in the preceding year. From January to March, a total of 63 cases of diphtheria were recorded.

Overall, the post-COVID-19 pandemic period witnessed a notable surge in diphtheria cases, with the age group of 0–10 years being predominantly affected. Surprisingly, there was no discernible trend regarding the dominance of diphtheria cases based on sex. Notably, despite the government's efforts in promoting vaccination programs across all regions, the available data highlights a concerning fact that a significant number of individuals remain unvaccinated against diphtheria. This underscores the importance of further initiatives and targeted measures to improve vaccination coverage.

### 3.2 Numerical simulation of mathematical model

**Fig. 5:**
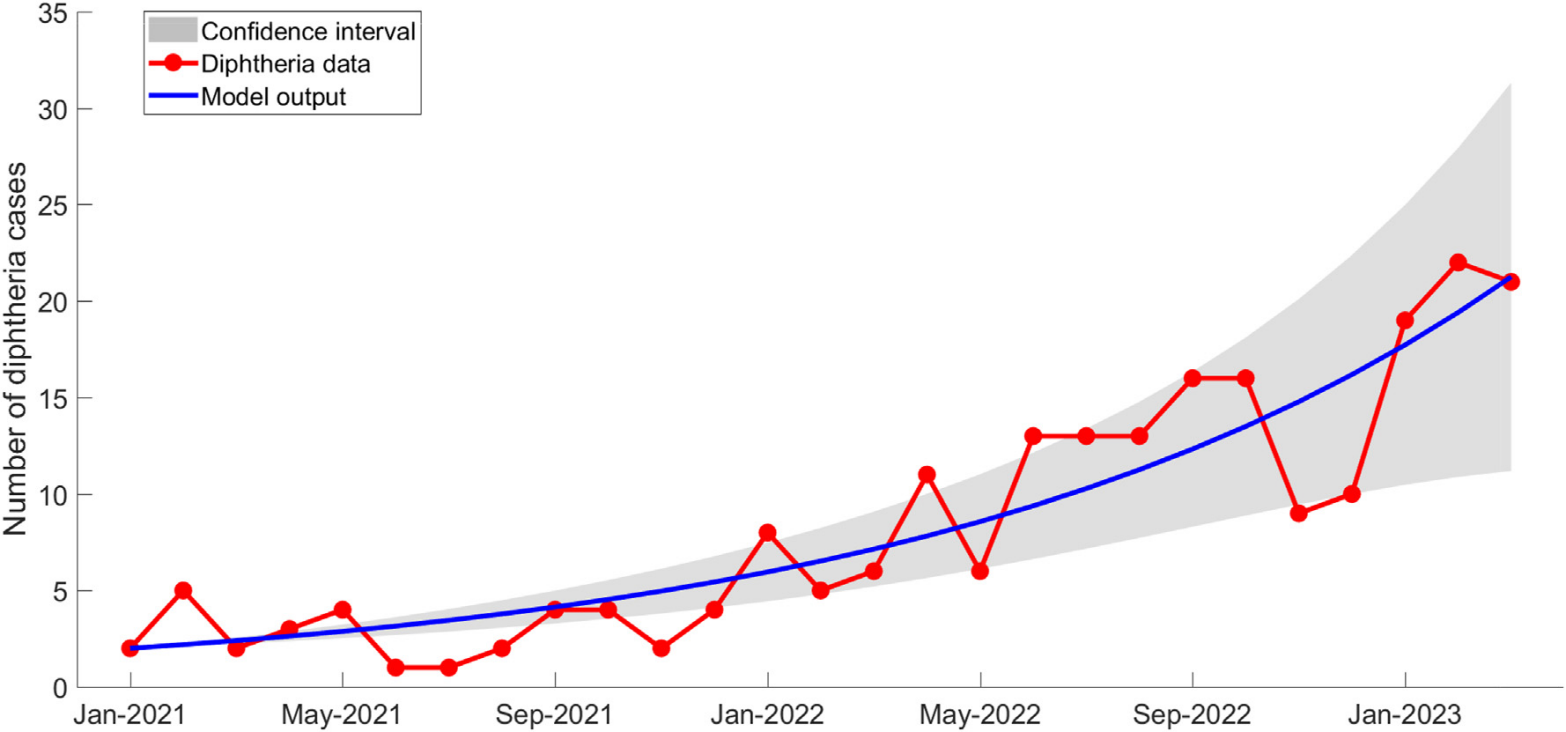
The simulation result of data fitting between diphtheria data and model output.

During the observation period, the monthly data of diphtheria cases was fitted to our mathematical model, and its output was utilized to determine the values of unobserved parameters, (*β*, *α*, *κ*). During the process of fitting data to a model, uncertainties may arise due to a lack of precision, one source of which is parameter uncertainty. The uncertainty in estimating parameters (*β*, *α*, *κ*) which are not precisely known, contributes to the overall uncertainty of the model fit. Addressing uncertainty is crucial for a comprehensive understanding of the reliability of the model and its predictions (Walker et al., 2003). Statistical measures such as confidence intervals can be utilized to quantify the uncertainty in these parameter estimates (Chowell, 2017). We employed bootstrap resampling of residuals (Carpenter & Bithell, 2000) to estimate the unobserved parameters, along with calculating the corresponding 95% confidence intervals. Table 3 displays the estimated values of each parameter and their associated confidence intervals resulting from 100 bootstrap realizations. The simulation results, presented with confidence intervals in Fig. 5, demonstrate a satisfactory fit to the actual data.

**Table 3:** Description of parameters used in mathematical model with estimated value.

Parameter	Description	95% Confidence Interval		
		**Estimated Value**	**Lower Bound**	**Upper Bound**
*β*	Infection rate	1.9927	1.9631	2.0223
*α*	Infection rate reduction constant	0.2634	0.2521	0.2747
*κ*	Probability of successful DPT vaccination	0.4426	0.4327	0.4524

We computed the Pearson correlation coefficient (denoted as *r*) between the actual data of diphtheria cases in West Java province and the output of the mathematical model to measure the linear relationship. The calculated correlation coefficient was found to be *r* = 0.9101 indicating a strong positive linear relationship between the data and the simulation outcome. To obtain a more robust assessment regarding the goodness-of-fit between data and predictions, we applied model validation using equivalence testing introduced by Robinson and Froese (Robinson & Froese, 2004), one of which is the paired *t*-test of equivalence. Equivalence testing reverses the common null hypothesis: they argue that the populations being compared are different and use the data to prove otherwise (Robinson et al., 2005). We set the hypothesis of dissimilarity as the null hypothesis, $H_{0}=\text{a difference, i.e.,}\;{\bar{x}}_{p}-{\bar{x}}_{m}\neq 0$, where $\bar{x}$ denotes mean, *p* refers to predicted, and *m* refers to measured. Using a paired *t*-test of equivalence at the significance level *α* = 0.05, we compared the calculated cutoff value and the obtained *t*-value. If the *t*-value was lower than the cutoff then we rejected the null hypothesis of dissimilarity (Robinson & Froese, 2004). The computed results revealed a cutoff value $C_{\alpha ;n-1}=4.237$, whereas the observed *t*-value was determined to be *t*_*d*_ = 0.126. Given that the calculated *t*-value falls below the specified cutoff value, we reject the null hypothesis of dissimilarity. This rejection implies that the model predictions are significantly similar to the actual data. The complete procedure for the equivalence test can be found in Appendix .3.

According to the simulation findings, the probability of the DPT vaccine effectively preventing diphtheria stands at approximately 44.26%, which is considerably lower than the real-world effectiveness, which ranges from 87% to 96% (Bedford & Elliman, 2000). Meanwhile, administering a booster vaccine decreases the infection risk by about 26.34% when compared to individuals who do not receive a booster. In addition, by using the estimated parameter values in Table 3, we can calculate the value of basic reproduction number (*R*_0_) for the current diphtheria cases in West Java. Basic reproduction number is generally known as the average number of secondary infections caused by an infected individual to the fully susceptible population in epidemiological models. The value of *R*_0_ holds significant importance as it helps predict whether a disease will disappear or continue to spread. By substituting estimated value (*β*, *α*, *κ*) and fixed value (*μ*, *γ*, *p*, *q*), we obtain the value of the basic reproduction number *R*_0_ ≈ 1.17. A value of *R*_0_ > 1 suggests that the diphtheria disease can sustain transmission within the population. As infected individuals continue to spread the infection to multiple susceptible individuals, the number of cases can increase exponentially over time. After cases have increased and reached a peak, the cases decrease gradually towards its endemic equilibrium. Therefore, based on the analysis of current epidemiological trends, it is evident that diphtheria cases are more likely of experiencing a surge in West Java as also supported by routine surveillance data.

Table 4 displays the sensitivity indices of *R*_0_ to each paramater for the diphtheria model. It's important to mention that this sensitivity analysis is a local sensitivity analysis, limited to the neighborhood of a point being assessed. A positive sensitivity index indicates that an increase in a parameter's value leads to an increase in *R*_0_, while a negative sensitivity index indicates that an increase in a parameter's value results in a decrease in *R*_0_. As can be seen in Table 4, the infection rate (*β*) has the highest sensitivity with *S*_*β*_ = +1.0000, implying that a 10% increase (decrease) in *β* corresponds to a 10% increase (decrease) in *R*_0_. The parameters *γ*, *κ*, *p*, and *q* also significantly affect *R*_0_, with *S*_*γ*_ = −0.9974, *S*_*κ*_ = −0.7745, *S*_*p*_ = −0.7745, and *S*_*q*_ = −0.9144. We highlighted the vaccination coverage parameters *p* (DPT) and *q* (booster) where a 10% increase in these parameters results in a decrease in the *R*_0_ value of around 7.745% and 9.144% respectively. The natural birth and mortality rate of humans (*μ*) is the least sensitive parameter to *R*_0_, where a 10% increase (decrease) in *μ* results in only a 0.026% decrease (increase) in *R*_0_. Since the infection rate (*β*) has the most significant effect on *R*_0_, it should be the focus when designing control strategies. Additionally, improvements in diphtheria treatment (*γ*), expansion of DPT vaccinations (*p*), and booster vaccinations (*q*) can also significantly reduce the value of *R*_0_, thus helping to prevent large outbreaks of diphtheria.

**Table 4:** Sensitivity indices of the basic reproduction number *R*_0_ to model parameters.

**Parameter**	*β*	*μ*	*γ*	*α*	*κ*	*p*	*q*
**Sensitivity index**	+1.0000	−0.0026	−0.9974	+0.3269	−0.7745	−0.7745	−0.9144

**Fig. 6:**
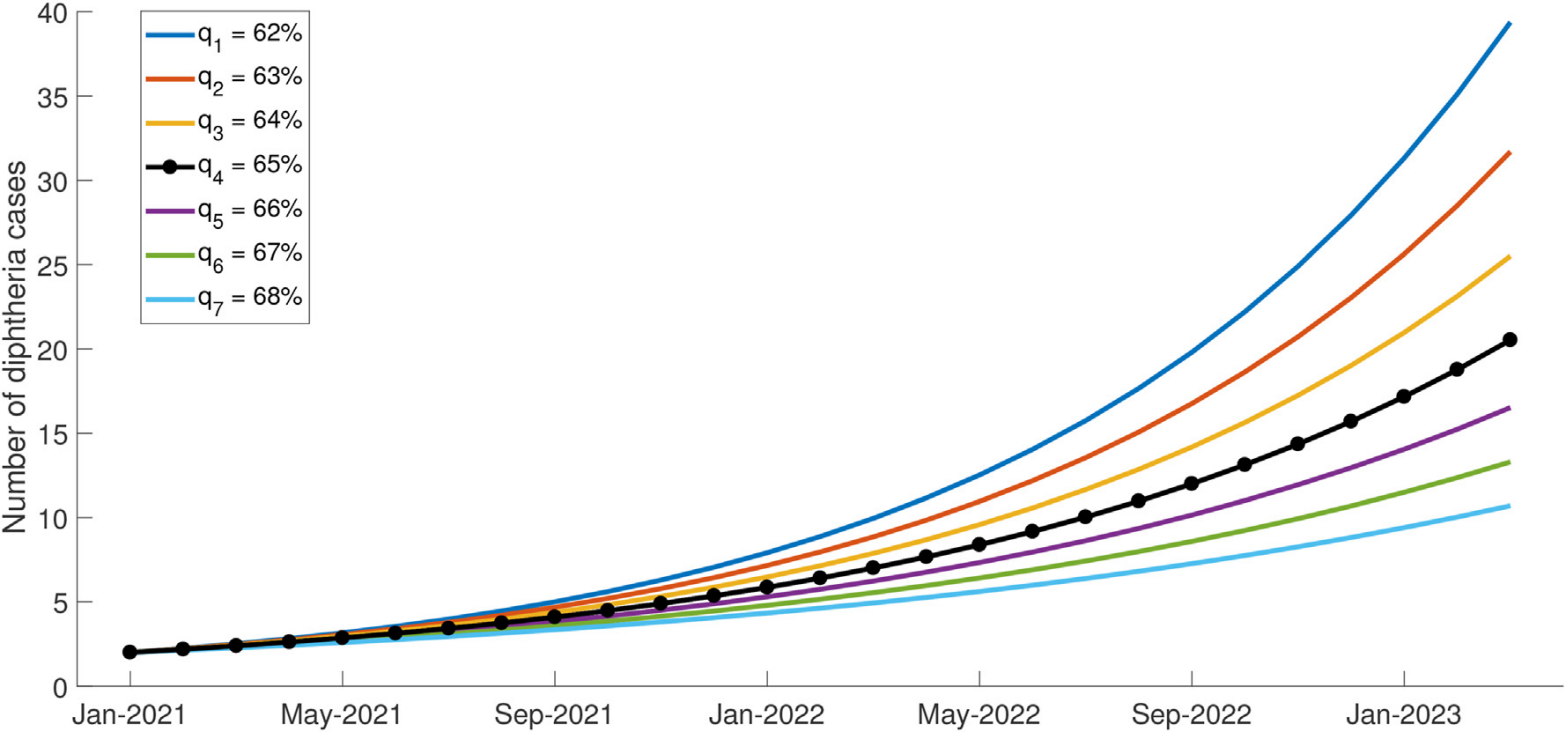
Variations of parameter *q* with 62% ≤ *q* ≤ 68% on the number of diphtheria cases. Black line with circles shows the current condition in West Java with a booster vaccination coverage of around 65%.

Booster vaccinations is generally recognized as playing a crucial role in significantly reducing the number of diphtheria cases within a population. While primary vaccinations are administered during childhood, the protective immunity can wane over time. By administering booster doses at recommended intervals, typically in adolescence or adulthood, individuals can maintain robust immunity against the bacterium, thereby preventing infections and limiting the spread of the disease. Booster vaccination coverage for diphtheria in West Java province is categorized as decent with an average percentage for the 2015–2022 period of 64.84% (see Fig. 2). We simulated numerically the effect of booster vaccination on the number of diphtheria incidents by varying the value of the parameter *q*. Fig. 6 presents the results of numerical simulations using various *q* values with 62% ≤ *q* ≤ 68%. It can be observed that the smaller the *q* value, the higher the number of diphtheria cases. Conversely, the greater the value of *q* the lower the potential number of diphtheria cases. The high upward trend at the beginning indicates that the peak of the outbreak occurred earlier and the number of cases at the peak was larger. The simulation results indicate that booster vaccination needs to be optimized in supporting the control of the spread of diphtheria and reducing the risk of the emergence of an outbreak.

**Fig. 7:**
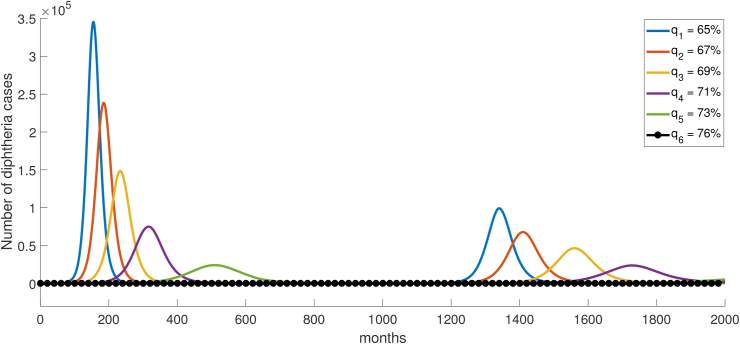
Variations in the coverage of booster vaccinations below and above the threshold (75.15%) for the number of diphtheria cases. A horizontal black line with circles denotes a decrease in cases towards disease-free equlibrium with a parameter value of *q* > 75.15%.

Booster vaccination coverage is also an important parameter that determines the value of the basic reproduction number. Eq. (4) highlights the direct impact of booster vaccination coverage on the value of *R*_0_, where the parameter *q* represents the proportion of the susceptible population covered by the booster vaccination. By considering disease-free scenarios with *R*_0_ < 1, it becomes possible to ascertain the maximum coverage limit of booster vaccinations required to prevent a surge in cases and avert the occurrence of an outbreak with a substantial number of infections. By solving the inequality *R*_0_ < 1, a solution of *q* > 75.15% is obtained. This implies that as long as the percentage of booster vaccination coverage remains below 75.15%, there exists a possibility of a diphtheria outbreak. The closer the vaccination coverage is to this critical threshold, the more delayed the peak of the outbreak will be. Conversely, if the vaccination coverage surpasses 75.15%, there is a likelihood of achieving a disease-free state, indicating the potential for the disease to be eradicated. Fig. 7 shows that the number of cases tend to disease-free equilibrium as the percentage of booster vaccination coverage is above 75.15%.

### 3.3 Risk zone mapping using hot spot analysis

**Fig. 8:**
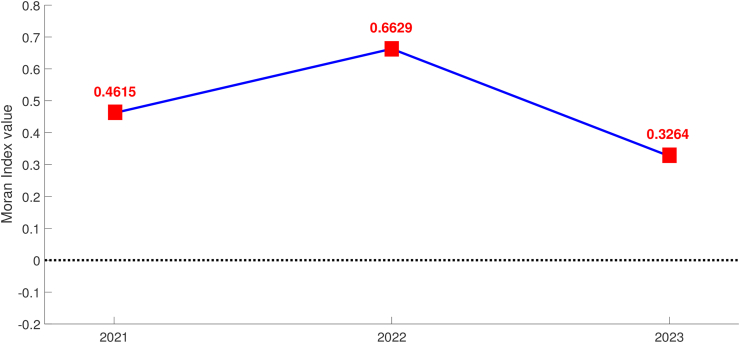
Moran's Index value of diphtheria cases in West Java during 2021–2023 period.

The value of Moran's index, a measure used to assess spatial autocorrelation, exhibited varying patterns over the years 2021, 2022, and 2023 as can be seen in Fig. 8. In general, a positively significant Moran's Index coefficient of diphtheria data was observed that indicates the existence of spatial clustering. In 2021, the index recorded a value of 0.4615, indicating a moderate level of spatial autocorrelation among the data points. The subsequent year, 2022, witnessed a significant increase in the Moran's index, reaching 0.6629, signifying a stronger spatial clustering or correlation among the observed variables. However, in 2023, there was a decline in the Moran's index value to 0.3264, indicating a weakening of spatial autocorrelation compared to the previous year. These changes in Moran's index values reflect the fluctuating spatial patterns and interdependencies among the data points over the three-year period. Understanding these trends can provide valuable insights for spatial analysis and decision-making processes in relevant domains.

**Fig. 9:**
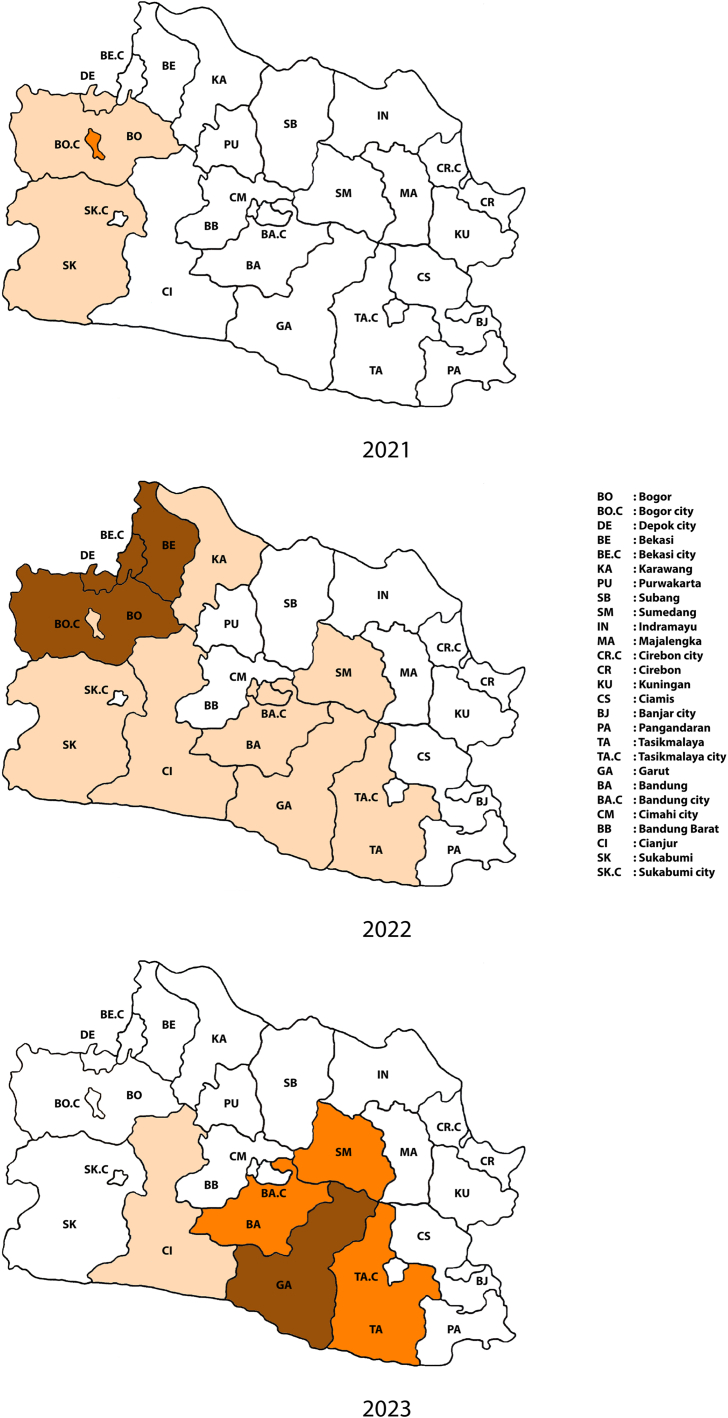
Hot spot analysis of diphtheria cases in West Java. High-level hot spots $\left(Z_{G_{i}^{*}}>2\right)$ were shown in brown color, moderate-level hot spots $\left(1<Z_{G_{i}^{*}}\leq 2\right)$ colored with orange, and low-level hot spots $\left(0<Z_{G_{i}^{*}}\leq 1\right)$ were indicated by beige color. Meanwhile, cold spots with $Z_{G_{i}^{*}}$ were shown in white color.

At the local level, the results of the analysis using $Z_{G_{i}^{*}}$ are shown in Fig. 9. The identification and visualization of hot spots and cold spots in the given dataset are based on the values of Z-scores. Hot spots with $Z_{G_{i}^{*}}>2$ are considered as high-level hot spot and are highlighted using a brown color. Alternatively, hotspots with $1<Z_{G_{i}^{*}}\leq 2$ are represented with an orange color, indicating a moderate-level hot spot. While these hot spots are statistically significant, the level is slightly lower compared to the first category. For $0<Z_{G_{i}^{*}}\leq 1$, a beige color is assigned to indicate a hot spot with low level and still statistically significant. On the other hand, negative Z-scores $\left(Z_{G_{i}^{*}}\leq 0\right)$ indicate cold spots, regions with values lower than expected, and come with not statistically significant.

In 2021, the identification of hot spots in West Java province revealed clustering primarily in the western region. Within this area, three specific regions, namely Depok city, Bogor district, and Sukabumi district, were designated as low-level hotspots. Bogor city is identified as a moderate-level hot spot colored with orange. However, the following year, 2022, presented unsatisfactory results as the number of hotspots increased significantly and dispersed across the western, central, and southern regions of West Java province. A total of 14 areas were identified as hotspots with varying levels, ranging from low to high, signifying intensified spatial clustering. The western region of West Java province, which was initially categorized as a low-level hotspot, underwent a significant change and transformed into a high-level hotspot, as indicated by the brown color on the map. Several areas within this region, including Depok city, Bogor district, Bekasi city, and Bekasi district, now fall into the category of high-level hotspots, signifying intensified spatial clustering. Notably, the western region is characterized by a dense population and forms part of the bustling metropolitan area of Jakarta, the capital of Indonesia, contributing to a massive level of population mobility. Several areas in the central and southern parts of the province were also identified as hotspots, albeit with low levels of significance. Bandung city, serving as the capital of West Java and boasting a significant population, along with Cimahi city, have been identified as low-level hot spots.

However, during early 2023, a shift occurred in the western region of West Java province, transforming it into a cold spot. Conversely, hot spots emerged in the southern and several regions in central areas of the province. In this period, several districts and cities that were originally classified as low-level hot spots (beige) in 2022 have now changed to orange or brown, reflecting increased significance in spatial clustering. Among these, Garut district stands out as a high-level hot spot, while Bandung district, Sumedang district, and Tasikmalaya district are identified as moderate-level hot spots. Notably, unlike the western and central regions, the southern areas, particularly Garut and Tasikmalaya districts, are characterized by villages with relatively low population densities.

Throughout the observation period, an absence of hot spots was noted in the northern and eastern regions of West Java province. Despite the fact that the northern region, along with certain parts of the eastern region, constitutes one of the densest industrial areas in the province, encompassing areas such as Karawang district, Purwakarta district, Subang district, and Cirebon district/city, it was intriguingly identified as a cold spot with a relatively low risk of diphtheria cases compared to other areas. The unexpected absence of hot spots in this industrial hub implies that industrialization alone may not be the primary driver of diphtheria transmission in these specific areas. The number of hot spots for each category throughout the observation time is presented in Table 5.

**Table 5:** The number of hot spots for each category during 2021–2023 period.

Year	Category		
	**High-level hotspot**	**Moderate-level hotspot**	**Low-level hotspot**
2021	0	1	3
2022	4	0	10
2023	1	3	1

Based on the classification of diffusion patterns introduced by Hornsby (Hornsby, 2011), the diffusion pattern of diphtheria cases in West Java exhibits a combination of contagious and expansion diffusion. The disease continues to spread outward from the initial location, creating spatial clusters or concentrations of cases. The spread of cases was initiated from the western region in West Java province and gradually expanded into the central and southern regions of the province. As the cases continue to propagate, the expansion diffusion component comes into play, resulting in the gradual dissemination of the disease to neighboring areas in the central and southern regions. This unique combination of diffusion mechanisms underscores the complex dynamics of diphtheria transmission in West Java and emphasizes the need for comprehensive surveillance and targeted intervention strategies to effectively manage the disease's spatial spread across the province.

## 4 Discussion

The COVID-19 pandemic has significantly impacted healthcare services worldwide, causing a burden on healthcare systems, disrupting non-emergency care, leading to shortages of medical supplies and personnel, and causing delayed diagnoses and treatments for other medical conditions. The diversion of healthcare resources and attention towards managing the pandemic has significantly strained healthcare systems, resulting in reduced access to essential preventive and curative services. Also, the pandemic has had a profound impact on immunization programs globally. Disruption of routine immunization services due to overwhelmed healthcare systems has left vulnerable populations susceptible to preventable diseases. Numerous studies have demonstrated that the disruption of health services during the COVID-19 pandemic has led to suboptimal healthcare delivery including prevention, diagnosis, and treatment for other diseases, such as tuberculosis (Alene et al., 2020), diphtheria (Chiappini et al., 2021), measles (Cioffi & Cecannecchia, 2022), hepatitis (Pley et al., 2021), and diabetes mellitus (Nuraini et al., 2022).

Regarding incidents of diphtheria, significant outbreaks of the disease have been frequently observed following interruptions in vaccination programs, as exemplified by occurrences in the ex-Soviet Union countries during the 1990s and more recent instances in Venezuela and the Rohingya refugee population in Bangladesh. The disruption induced by pandemic has a crucial impact on immunization programs and the treatment of diphtheria, potentially leading to the re-emergence or an increase in the number of diphtheria cases worldwide. This study reveals a notable upward trend in diphtheria cases in West Java with the value of *R*_0_ ≈ 1.17 following the COVID-19 pandemic. The *R*_0_ value exceeding 1 indicates the potential for continued escalation of diphtheria cases in the region, necessitating prompt and targeted interventions from the local government and relevant authorities to effectively control the outbreak. Similar research findings were observed in Pakistan, India, Nigeria, and Peru. The study by Saeed et al. (Saeed et al., 2023) revealed an increase in diphtheria cases after the COVID-19 pandemic in Pakistan, resulting in the tragic loss of over 45 children and teenagers in 2022, with numerous suspected cases reported from various regions. In northwestern Nigeria, Ibrahim et al. (Ibrahim et al., 2022) reported 35 diphtheria cases in 2020, with a case fatality rate of up to 68.6%. Additionally, the World Health Organization (WHO) confirmed more than 3000 cases of diphtheria across India (World Health Organization, 2023). Peru also experienced the re-emergence of diphtheria, with the first reported case in two decades, suspected to be linked to the government's lockdown measures during the pandemic (Mezones-Holguin et al., 2021).

Booster vaccination plays a crucial role in controlling diphtheria outbreaks, especially when coupled with primary vaccination using the DPT (diphtheria, pertussis, and tetanus) vaccine. Boosters help reinforce the body's immune response, providing additional protection against diphtheria and extending immunity beyond the initial vaccination period. The DPT vaccine, administered during childhood, lays the foundation for immunity; however, over time, immunity may wane, making booster shots essential to maintain long-term protection. While the inclusion of diphtheria in the DPT vaccine has significantly reduced diphtheria incidence worldwide, the global status of booster vaccination for diphtheria varies among countries. Some countries have robust booster vaccination programs, ensuring continuous protection, while others may face challenges in achieving high vaccine coverage. In the European region, each of the 53 countries administered a minimum of one booster dose, and 37 countries adhered to the guideline of administering three or more recommended booster doses (Muscat et al., 2022). The study conducted in Asia emphasizes the insufficiencies in immunity, attributed to gaps in recommendations or inadequate booster coverage, underscoring the necessity for booster vaccinations in this population to address public health concerns (Nicholson et al., 2022). In contrast, certain African countries, including Nigeria, encounter challenges in attaining and maintaining high vaccination coverage due to frequent unavailability of vaccines (Shariff et al., 2023). Within our study area, the DPT 1–3 vaccination coverage exhibited a commendable high average of 98.62% during the period 2015–2020. Meanwhile, the coverage for booster vaccination remained at a modest 64.84%. Simulation results demonstrate that boosting vaccination rates to 75.15% would be pivotal in effectively controlling diphtheria and preventing extensive outbreaks in the region.

In our study, we observed that the regions with high-risk of diphtheria infection were scattered across areas exhibiting varying population densities, ranging from low to high. Spatial analysis initially identified hotspots in the western region, characterized by densely populated areas. Subsequently, these hot spots expanded to the surrounding regions through diffusion. The diffusion of the diphtheria phenomenon can be classified as an expansion-contagious type, where it spread from the western region to the central and southern regions. The western region, comprising urban areas like Depok, Bekasi, and Bogor, is densely populated, with a population density exceeding 9,000 individuals/km^2^. High mobility in this region can be attributed to its proximity to the capital city of Jakarta. Although the risk of diphtheria is higher in densely populated urban areas, there is still a possibility of outbreaks occurring in rural areas, as evidenced by occurrences in southern region such as Garut, Tasikmalaya, Cianjur, Sumedang, and Sukabumi districts. The population density within this area amounts to fewer than 1,000 individuals/km^2^.

Multiple research findings indicate a tendency for diphtheria outbreaks to occur in densely populated areas. For instance, Strauss et al. (Strauss et al., 2021) documented that in Venezuela, a significant number of diphtheria cases during the period 2016–2019 were concentrated in hospitals located in densely populated states. Similarly, research conducted by Saito et al. (Saito et al., 2021) in the Philippines revealed higher occurrences of diphtheria cases in densely populated regions between 2006 and 2017, although no apparent clustering of specific ST types was observed. Moreover, Adegobye et al. (Adegoboye et al., 2023) reported the resurgence and re-emergence of diphtheria in Nigeria during 2023, primarily in highly densely populated areas. It is essential to note that rural areas are not exempt from the risk of diphtheria cases, as evidenced in Vietnam and India. According to the research conducted by Kitamura et al. (Kitamura et al., 2023), there have been multiple small diphtheria outbreaks in remote districts of central and western Vietnam since 2013. Similarly, the study by Shetty et al. (Shetty et al., 2020) found that 16 out of 18 diphtheria cases in South India originated from rural areas.

This study possesses several limitations that offer opportunities for further development. The initial limitation pertains to data availability, which currently remains incomplete, particularly concerning vaccination details of infected individuals. Acquiring such information is crucial to assess the effectiveness of vaccination in preventing diphtheria infections. Additionally, the possibility of unrecorded diphtheria cases exists due to constraints in data recording. The second limitation is associated with the mathematical model employed. The model utilized in this study is relatively simplistic, involving only the SIR compartment. To address this limitation, more sophisticated models could be formulated, encompassing various real-world phenomena in diphtheria transmission by incorporating additional compartments for both complete and partial vaccinations.

## 5 Conclusions

In summary, this research paper presents a mathematical model that combines diphtheria vaccination data, and describes a spatial analysis of diphtheria cases in West Java. The disruptions caused by the COVID-19 pandemic have negatively affected vaccination efforts and the management of diphtheria cases, leading to a rise in diphtheria cases after the pandemic. The mathematical model calculates a basic reproduction number greater than 1, indicating the potential for diphtheria outbreaks, but these can be prevented by improving booster vaccination coverage. Spatial analysis reveals high-risk areas in the western, central, and southern regions of West Java. These regions are not only densely populated but also consist of rural areas with low population density. Consequently, local governments and health authorities should take these findings into account to prevent large diphtheria outbreaks and devise effective strategies to safeguard the population.

## CRediT authorship contribution statement

**Ilham Saiful Fauzi:** Writing – original draft, Software, Methodology, Investigation, Formal analysis, Data curation, Conceptualization. **Nuning Nuraini:** Writing – review & editing, Supervision, Investigation, Funding acquisition, Conceptualization. **Ade Maya Sari:** Software, Investigation. **Imaniah Bazlina Wardani:** Writing – original draft, Investigation. **Delsi Taurustiati:** Supervision, Resources. **Purnama Magdalena Simanullang:** Supervision, Resources. **Bony Wiem Lestari:** Writing – review & editing, Supervision, Investigation, Formal analysis.

## Declaration of competing interest

The authors declare that they have no known competing financial interests or personal relationships that could have appeared to influence the work reported in this paper.

## Figures and Tables

**Table 6:** The data of diphtheria cases and model prediction throughout the observation period.

Period	Prediction	Measured Data	Difference
2021–January	2.000	2	0.000
2021–February	2.191	5	−2.809
2021–March	2.399	2	0.399
2021–April	2.628	3	−0.372
2021–May	2.878	4	−1.122
2021–June	3.152	1	2.152
2021–July	3.452	1	2.452
2021–August	3.781	2	1.781
2021–September	4.141	4	0.141
2021–October	4.535	4	0.535
2021–November	4.967	2	2.967
2021–December	5.440	4	1.440
2022–January	5.957	8	−2.043
2022–February	6.524	5	1.524
2022–March	7.145	6	1.145
2022–April	7.825	11	−3.175
2022–May	8.570	6	2.570
2022–June	9.385	13	−3.615
2022–July	10.278	13	−2.722
2022–August	11.255	13	−1.745
2022–September	12.326	16	−3.674
2022–October	13.498	16	−2.502
2022–November	14.782	9	5.782
2022–December	16.187	10	6.187
2023–January	17.727	19	−1.273
2023–February	19.412	22	−2.588
2023–March	21.257	21	0.257

## Appendix.1The Derivation of the Basic Reproduction Number *R*_0_

The calculation of the basic reproduction number *R*_0_ was accomplished through the application of the Next Generation Matrix (NGM). We only considered the disease compartment. Let *x* = *I*, we determined which terms from the equation in the disease compartment represent new infections (denoted as F), and which terms represent movement among or out of the disease compartment (denoted as V).

$$F=\left[\beta \left(1-q\right)+\alpha \beta q\right]\left(N-\left(I+R\right)\right)\frac{I}{N}\;\;\;\;V=\left(\gamma +\mu \right)I$$

Then, we took the derivative of F and V respect to the state variable of the disease compartment, *x*. We called the resulting matrices *F* and *V*.

$$F=\frac{\partial F}{\partial x}=\left[\beta \left(1-q\right)+\alpha \beta q\right]\frac{\left(N-\left(2I+R\right)\right)}{N}\;\;\;V=\frac{\partial V}{\partial x}=\gamma +\mu$$

Using normalization on the *N* value where *N* = 1, we evaluated *F* and *V* at the DFE (disease-free equilibrium) i.e., (*I*°, *R*°) = (0, *κpN*) = (0, *κp*). Hence, we acquired:

$$F{|}_{I{}^{\circ},R{}^{\circ}}=\left[\beta \left(1-q\right)+\alpha \beta q\right]\left(1-\kappa p\right)\;\;V{|}_{I{}^{\circ},R{}^{\circ}}=\gamma +\mu$$

We obtained the next generation matrix as follows:

$$FV^{-1}=\left[\frac{\left[\beta \left(1-q\right)+\alpha \beta q\right]\left(1-\kappa p\right)}{\gamma +\mu }\right]$$

The basic reproduction number was then derived from the dominant eigenvalue of the *FV*^−1^ matrix and shown as follows:

(1) $$R_{0}=\rho \left(FV^{-1}\right)=\frac{\beta \left(1-q+\alpha q\right)\left(1-\kappa p\right)}{\gamma +\mu }$$

## Appendix.2 The Stability Analysis of Equilibriums

Theorem Appendix .1. If *R*_0_ < 1, then DFE is locally asymptotically stable.

*Proof*. The Jacobian matrix at the disease-free equilibrium is given by:

(2) $$J{}^{\circ}=\left[\begin{array}{cc} \delta \left(1-\kappa p\right)-\left(\mu +\gamma \right) & 0\\ \gamma & -\mu \end{array}\right]$$

The characteristic polynomial of matrix *J*° is *P*(*λ*) = *λ*^2^ + *a*_1_*λ* + *a*_2_, with:

$$\begin{array}{ll} & a_{1}=\mu -\left(\mu +\gamma \right)\left(R_{0}^{2}-1\right)\\ & a_{2}=-\mu \left(\mu +\gamma \right)\left(R_{0}^{2}-1\right) \end{array}$$

Since all parameters are positive, then *R*_0_ < 1 implies *a*_1_, *a*_2_ > 0. Based on *Routh-Hurwitz*'s stability criterion, the polynomial has roots with a negative real part. Therefore, the disease-free equilibrium (DFE) is locally asymptotically stable. □

Theorem Appendix .2. If *R*_0_ > 1, then EE is locally asymptotically stable.

*Proof*. The Jacobian matrix at the endemic equilibrium is given by:

(3) $$J^{*}=\left[\begin{array}{cc} \delta \left(1-2\varphi -\psi \right)-\left(\mu +\gamma \right) & -\delta \varphi \\ \gamma & -\mu \end{array}\right]$$

with:

$$\varphi =\frac{\mu \left(\delta \left(1-\kappa p\right)-\left(\mu +\gamma \right)\right)}{\delta \left(\mu +\gamma \right)}\;\;\psi =\frac{\delta \left(\kappa p\mu +\gamma \right)-\gamma \left(\mu +\gamma \right)}{\delta \left(\mu +\gamma \right)}\;\;\delta =\beta \left(1-q+\alpha q\right)$$

The characteristic polynomial of matrix *J*∗ is *P*(*λ*) = *λ*^2^ + *a*_1_*λ* + *a*_2_, where:

$$\begin{array}{ll} & a_{1}=\mu \left(R_{0}^{2}-1\right)+\mu \\ & a_{2}=\mu \left(\mu +\gamma \right)\left(R_{0}^{2}-1\right) \end{array}$$

The value of parameter *μ* and *γ* are positive. If *R*_0_ > 1, we obtain *a*_1_, *a*_2_ > 0. Considering the *Routh-Hurwitzs* stability criterion, the roots of polynomial *P*(*λ*) have negative real part. Hence, the endemic equilibrium (EE) is locally asymptotically stable. □

## Appendix.3 The Equivalence Testing

Here, we present model validation based on statistical inference that seeks to establish a statistical comparison of model predictions against the actual data. We implemented the equivalence testing with the paired *t*-test introduced by Robinson and Froese (Robinson & Froese, 2004) to assess the goodness-of-fit between data and the model output. We used the hypothesis of dissimilarity as the null hypothesis.

$$H_{0}=\text{a difference, i.e.,}\;{\bar{x}}_{p}-{\bar{x}}_{m}\neq 0$$

where $\bar{x}$ denotes mean, *p* refers to predicted, and *m* refers to measured.

The number of data pairs is *n* = 27. We calculated the mean of the difference ${\bar{x}}_{d}={\bar{x}}_{p}-{\bar{x}}_{m}=0.063$, standard deviation $s_{x_{d}}=2.598$, and standard errors $s_{{\bar{x}}_{d}}=s_{x_{d}}/\sqrt{n}=0.5$. We next determined the non-centrality parameter *ψ*^2^ by dividing the effect size by the variance scale factor. The effect size can be calculated based on the means of the groups being compared, and the variance scale factor is often related to the pooled variance. Here, we obtained *ψ*^2^ = 0.0001. Using significance level *α* = 0.05, we determined the cutoff $C_{\alpha ;n-1}$ of the non-central F distribution with degrees of freedom *ν*_1_ = 1 and *ν*_2_ = *n* − 1, and non-centrality parameter *ψ*^2^. The cutoff calculation results in $C_{\alpha ;n-1}=4.237$. We finally calculated the *t*-value corresponding to the observed mean and standard deviation by $t_{d}={\bar{x}}_{d}/s_{{\bar{x}}_{d}}=0.126$ and compared it with the cutoff. Since the *t*-value was lower than the cutoff then we rejected the null hypothesis of dissimilarity. The rejected null hypothesis implies that the model predictions are significantly similar to the actual data.
